# Antimicrobial and antibiofilm potential of biosurfactants isolated from lactobacilli against multi-drug-resistant pathogens

**DOI:** 10.1186/1471-2180-14-197

**Published:** 2014-08-14

**Authors:** Karthik Sambanthamoorthy, Xiaorong Feng, Ruchi Patel, Sneha Patel, Chrysanthi Paranavitana

**Affiliations:** 1 Department of Wound Infections, Bacterial Diseases Branch, Walter Reed Army Institute of Research, 503 Robert Grant Avenue, Silver Spring, MD 20910, USA

**Keywords:** Anti-adhesive, Anti-biofilm, Biosurfactant, Antimicrobial, *Lactobacillus jensenii*, *Lactobacillus rhamnosus*

## Abstract

**Background:**

Biosurfactants (BS) are amphiphilic compounds produced by microbes, either on the cell surface or secreted extracellularly. BS exhibit strong antimicrobial and anti-adhesive properties, making them good candidates for applications used to combat infections. In this study, our goal was to assess the *in vitro* antimicrobial, anti-adhesive and anti-biofilm abilities of BS produced by *Lactobacillus jensenii* and *Lactobacillus rhamnosus* against clinical Multidrug Resistant (MDR) strains of *Acinetobacter baumannii*, *Escherichia coli*, and *Staphylococcus aureus* (MRSA). Cell-bound BS from both *L. jensenii* and *L. rhamnosus* were extracted and isolated. The surface activities of crude BS samples were evaluated using an oil spreading assay. The antimicrobial, anti-adhesive and anti-biofilm activities of both BS against the above mentioned MDR pathogens were determined.

**Results:**

Surface activities for both BS ranged from 6.25 to 25 mg/ml with clear zones observed between 7 and 11 cm. BS of both *L. jensenii* and *L. rhamnosus* showed antimicrobial activities against *A. baumannii*, *E. coli* and *S. aureus* at 25-50 mg/ml. Anti-adhesive and anti-biofilm activities were also observed for the aforementioned pathogens between 25 and 50 mg/ml. Finally, analysis by electron microscope indicated that the BS caused membrane damage for *A. baumannii* and pronounced cell wall damage in *S. aureus*.

**Conclusion:**

Our results indicate that BS isolated from two Lactobacilli strains has antibacterial properties against MDR strains of *A. baumannii, E. coli* and MRSA. Both BS also displayed anti-adhesive and anti-biofilm abilities against *A. baumannii*, *E. coli* and *S. aureus*. Together, these capabilities may open up possibilities for BS as an alternative therapeutic approach for the prevention and/or treatment of hospital-acquired infections.

## Background

Biosurfactants (BS) are amphiphilic compounds produced mostly by microbes on their cell surface, or secreted extracellularly and exhibit strong surface and emulsifying activities. They contain both hydrophobic and hydrophilic moieties that can reduce the surface or interfacial tension in liquids [1]. BS are complex molecules that include glycolipids, rhamnolipids, lipopeptides, polysaccharide-protein complexes, phospholipids, fatty acids and neutral lipids [2]. Unlike synthetic surfactants, BS are diverse and biodegradable, and have the potential for highly selective, specialized functions. Several BS exhibit anti-bacterial, anti-fungal and anti-viral activities, making them appropriate candidates to combat infections [3].

The list of known BS includes surfactin, the most powerful BS known, which is produced by *Bacillus subtilis*[4]. Other BS with antimicrobial activity include iturin, also produced by *B. subtilis*[4], mannosylerythritol lipids from *Candida antarctica*[5], rhamnolipids from *Pseudomonas aeruginosa*[6] and those isolated from probiotic bacteria *Streptococcus thermophilus A* and *Lactococcus lactis*[7-9]. Probiotic lactobacilli, which constitute an important part of natural microbiota, are recognized as potent interfering bacteria due to the production of various antimicrobial agents including BS [10]. In one study, 15 Lactobacillus strains were tested *in vitro* for BS production. It was found that all released surface active components during their mid-exponential and stationary growth phases [6].

Another valuable attribute of BS is their use as anti-adhesive/anti-biofilm agents [3,11] as shown previously in the lack of adhesion of *Enterococcus faecalis* to glass with an adsorbed BS layer from *Lactobacillus acidophilus* RC14 or *Lactobacillus fermentum* B54 [12].

Biofilms are conglomerations of bacterial cells protected by self-synthesized extracellular polysaccharide matrices (EPS). Biofilm infections are extremely challenging to treat because antimicrobials are less effective than planktonic cells [13,14], thus making clearance more challenging. The presence of biofilms causes numerous problems in the field of medicine, interfering with clinical therapy of chronic and wound-related infections as well as persistent infections of various indwelling medical devices [15]. Although numerous strategies have been established and are currently in use to control biofilms, the pursuit for novel, natural, and effective anti-biofilm agents still continues [16-18].

In recent years, the use of BS as alternatives to control biofilms has been explored extensively [19-21]. BS have been shown to modify the surface properties of bacterial cells and reduce their adhesive properties [4]. In addition, BS produced by bacteria have been shown to interfere with biofilm development and cell to cell communication [22-24].

Studies in the past have demonstrated the ability of probiotic bacteria *L. acidophilus*-derived BS to inhibit staphylococcal biofilm development and also induce its dispersion [25]. BS produced by probiotic lactobacilli have been shown to reduce adhesion of pathogenic bacteria to glass, silicone rubber, surgical implants, and voice prostheses [8,12,26,27]. It is believed that when BS is applied to a substratum surface, it modifies its hydrophobicity, interfering in the microbial adhesion and desorption processes [28,29]. Consequently, prior application of BS on catheters and other medical insertion materials may be used as a preventive strategy to delay the onset of pathogenic biofilm growth of MDR bacteria on wounds, medical insertion materials and inert surfaces in the hospital environment [8,12,25-27,30]. Thus, the prevention of biofilm formation or disruption by natural lactobacilli-derived agents was tested in these *in vitro* studies as a possible approach leading to novel antimicrobials.

The aims of this study were to determine the antimicrobial, anti-adhesive, and anti-biofilm activities of cell-bound BS isolated from *L. jensenii* and *L. rhamnosus* against several clinical isolates of multidrug-resistant pathogens.

## Results

### Oil spreading assay

The oil spreading assay was utilized to study the surface activities of crude BS. This assay is rapid, and is highly sensitive to surface active compounds [31,32]. Both *L. jensenii* and *L. rhamnosus* strains demonstrated oil displacement activity in motor oil. The oil displacement activity, as measured by the area of the clear zone on the oil-water surface, increased with an increase in the concentration of BS (Table 1).

**Table 1:** Diameter (mm) of clearing zones on the oil surface obtained from oil spreading assay with different concentrations of crude biosurfactant

**Sample**	**PBS**	**Surfactin**	**Crude biosurfactant**	**Crude biosurfactant**
			***L. rhamnosus***	***L. jensenii***
50 mg/ml	0.0	15.0 ± 1.0	6.8 ± 0.5	7.6 ± 0.5
25 mg/ml	0.0	15.0 ± 1.0	6.0 ± 1.4	6.0 ± 1.7
12.5 mg/ml	0.0	14.6 ± 0.5	5.3 ± 0.5	5.6 ± 1.1
6.25 mg/ml	0.0	13.0 ± 0.5	4.1 ± 0.1	3.8 ± 0.2

PBS was used as a negative control with a clearing zone diameter of 0.0 mm. Surfactin was used as a positive control. Results shown are the average of ± SD of three experiments performed in triplicate.

### Antimicrobial assay with BS

The crude BS of both *L. jensenii* and *L. rhamnosus* were tested against two clinical isolates of MDR *A. baumannii*, *E. coli* and *S. aureus.* We found both BS to be effective in killing all three MDR pathogens at 50 mg/ml (Table 2). *L. jensenii* BS exhibited almost 100% activity against all the strains tested (Table 2). The activity of *L. rhamnosus* ranged from 96-97% against *A. baumannii* and 72-85% against *E. coli.* For *S. aureus* strains UAMS-1 and MRSA respectively, activity was between 80 and 93% respectively (Table 2).

**Table 2:** **Antimicrobial activity of the crude biosurfactant from *L. jensenii *** **(BSLJ) and *L. rhamnosus *** **(BSLR) against MDR pathogens**

	**AB5075**	**AB5711**	**EC438**	**EC433**	**MRSA**	***S. aureus*** **UAMS-1**	**KP4640**
BSLJ	99.0	100.00	99.00	99.00	99.0	99.0	99.9
SD	0.01	0	0.05	0.43	0.03	0.04	0.02
BSLR	96.35	97.85	72.34	85.34	93.27	80.54	91.6
SD	2.96	2.16	2.36	6.21	3.15	7.11	1.96

Results are from 3 independent preparations of BS. (AB = *A. baumannii*; EC = *E. coli*; MRSA = methicillin resistant *S. aureus*, KP = *Klebsiella pneumoniae* and SD = Standard deviation). Biosurfactants were tested at 50 mg/ml concentration. Numbers are represented and percent reduction in cell numbers when treated with BS.

### Impact of BS on bacterial attachment to abiotic surface

**Figure 1:**
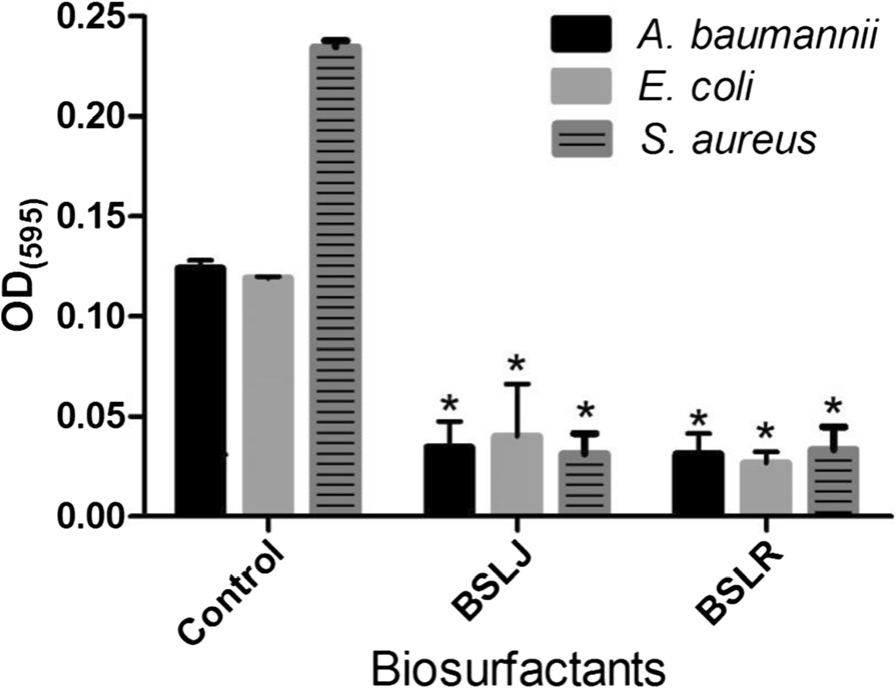
**The ability of biosurfactant from *L. jensenii *(BSLJ) and *L. rhamnosus *(BSLR) to reduce initial adherence of *A. baumannii, E. coli * and *S. aureus *is indicated.** BS was used at 50 mg/ml for *A. baumannii, E. coli* and at 25 mg/ml for *S. aureus.* Treated cells were statistically different from controls. The results represent the means plus SEM. Student’s paired *t* test was used to determine the statistical significance of the treated versus untreated conditions (*, *P* < 0.05).

Biofilm formation is a complex process that generally involves three stages: (1) primary adhesion to surfaces, (2) accumulation of multilayered clusters of cells, and (3) detachment. Because binding of host proteins is a major contributor to primary adhesion, it was important to test initial adherence to surfaces that were coated with plasma. Experiments were performed to determine the stage at which BS disrupts biofilm formation. Using an adherence assay, the ability of two BS to inhibit the cell attachment in the presence of host proteins was measured by coating the plates with human plasma. After various concentrations of BS ranging from 25-50 mg/ml were tested, it was found that the two BS significantly impaired the attachment of *A. baumannii* and *E. coli* at 50 mg/ml (Figure 1). *S. aureus* adherence to abiotic surfaces was disrupted at concentrations between 25 and 50 mg/ml (Figure 1).

### Impact of BS on biofilm development

**Figure 2:**
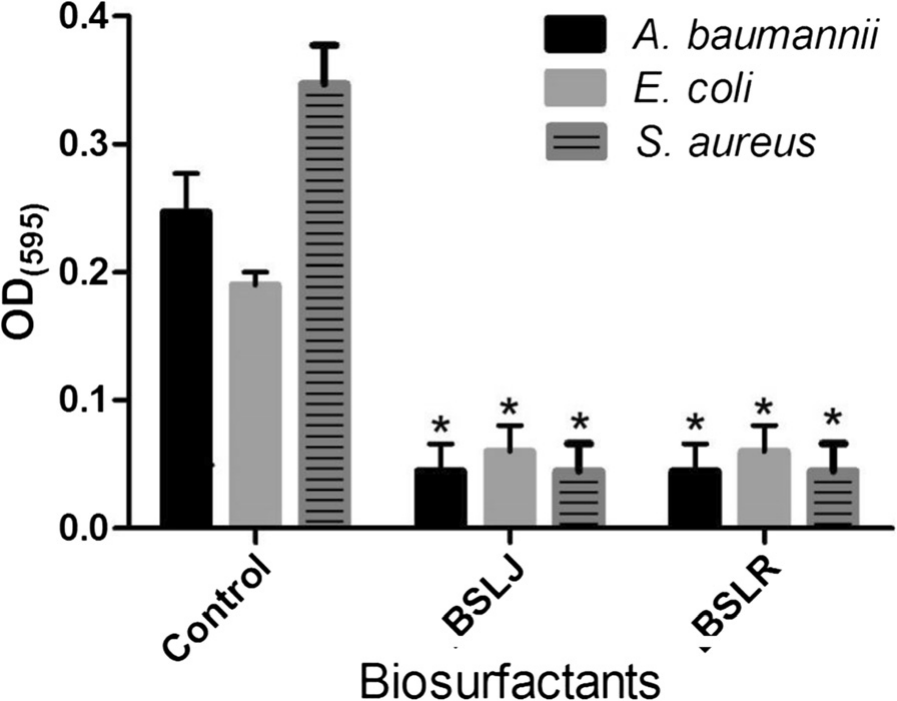
**The ability of biosurfactants from *L. jensenii *(BSLJ) and *L. rhamnosus *(BSLR) in reducing biofilm formation in *A. baumannii, E. coli * and *S. aureus *is indicated.** BS was used at 50 mg/ml for *A.baumannii*, *E.coli* and at 25 mg/ml for *S. aureus*. Treated cells were statistically different from controls. The results represent the means plus SEM. Student’s paired *t* test was used to determine the statistical significance of the treated versus untreated conditions (*, *P* < 0.05).

Next, it was determined if the static biofilm assay could show whether both BS possessed anti-biofilm activity against *A. baumannii*, *E. coli* and *S. aureus*. The BS produced by both *L. jensenii* and *L. rhamnosus* significantly reduced biofilm development by *A. baumannii* and *E. coli* at 50 mg/ml (Figure 2). Furthermore, the two BS significantly inhibited *S. aureus* biofilms at both concentrations of 25 mg/ml respectively (Figure 2).

### Dispersion of preformed biofilms by BS

**Figure 3:**
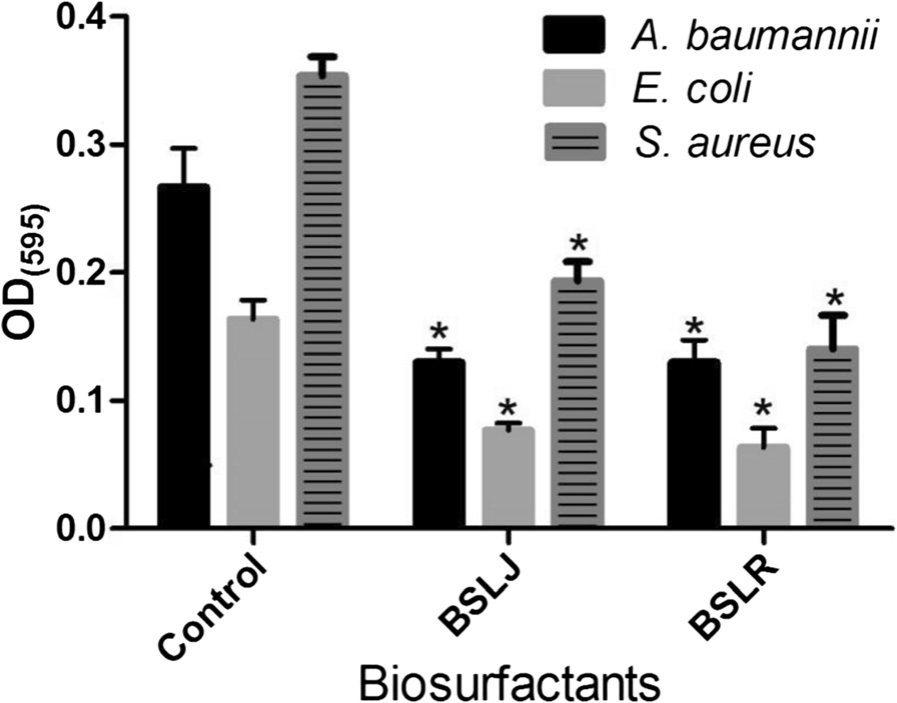
**The ability of biosurfactants from *L. jensenii *(BSLJ) and *L. rhamnosus *(BSLR) in dispersing biofilm formation in *A. baumannii, E. coli * and *S. aureus *is indicated.** BS was used at 100 mg/ml for *A. baumannii, E. coli* and at 50 mg/ml for *S. aureus.* Treated cells were statistically different from controls. The results represent the means plus SEM. Student’s paired *t* test was used to determine the statistical significance of the treated versus untreated conditions (*, *P* < 0.05).

In the experiments thus far, the BS were added concurrently with inoculation of bacteria. To determine if these compounds dispersed preformed biofilms, *A. baumannii* and *E. coli* biofilms were developed on MBEC pegs, and then exposed to varying concentrations of BS in fresh media for varying time intervals. After removal of the pegs, the amounts of bacteria that remained on the pegs were quantified by crystal violet staining. Compared to the controls, preformed *A. baumannii* and *E. coli* biofilms treated with BS at 100 mg/ml for 1 hr did not produce any dispersion effect. However, when biofilms were exposed for longer durations (~18 hrs), an increased biofilm dispersion was observed (Figure 3). Furthermore, an increase in dispersion was also observed for *S. aureus* biofilms when exposed to the BS at concentrations of 50 mg/ml for 18 hrs respectively (Figure 3).

### Cytotoxicity assays for BS in eukaryotic cells

**Figure 4:**
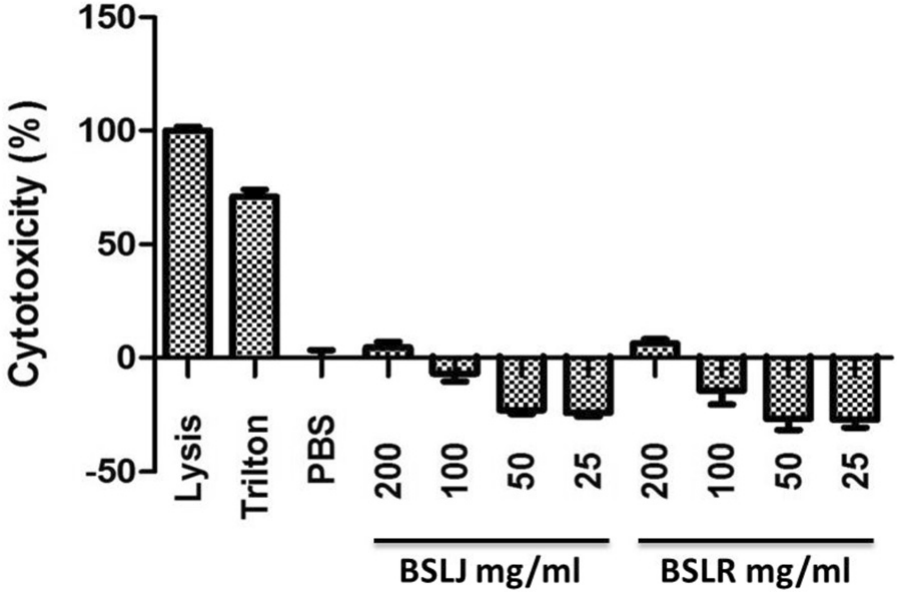
**Toxicity testing of BS in mammalian cells.** A549 lung epithelial cells were treated as indicated and viability was measured with LDH assay at 24 hours by following manufacturer’s direction. X-axis indicates the BS, positive (triton) and negative (PBS) controls.

Results from the cell-mediated cytotoxicity assay are presented in Figure 4. Human A549 lung epithelial cells were treated with BS from both *Lactobacilli* strains at various concentrations (25, 50, 100 and 200 mg/ml) for 24 h. Cytotoxicity was determined by LDH release according to the manufacturer’s instructions and the total cell number assay (Figure 4). Concentrations of 25-100 mg/ml showed no toxicity. Both BS showed very low toxicity levels at 200 mg/ml (Figure 4).

### Transmission electron microscopy

**Figure 5:**
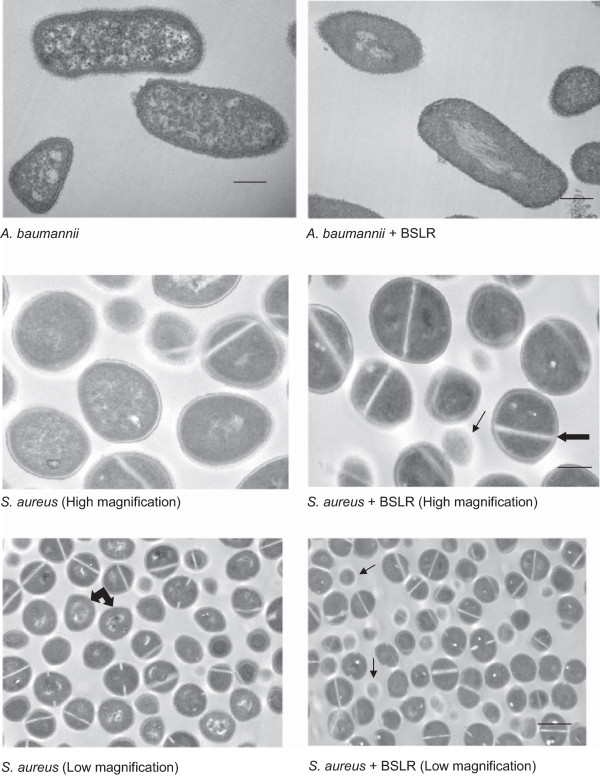
**Transmission electron microscope details on *A. baumanni *and *S. aureus *treated with BSLR.***A. baumannii* and *S. aureus* grown in the presence and absence of BSLR (4X MIC) were imaged at 3 h post incubation. Photographs were taken at a magnification of × 15000. The thick arrow indicates septum formation and the thin arrow indicates the ghost cells appearance respectively. For *S. aureus,* images were also acquired at lower magnification of ×8000, where a visible difference was seen between treated and untreated samples. BSLR treated *S. aureus* showed many more ghost cells (thin arrows) and cells without visible intracellular organelles compared to untreated cells (thick double arrows).

TEM was used to evaluate the ultra-structural morphological alterations exerted to *A. baumannii* and *S. aureus* in the presence of BSLR. We chose *A. baumannii* 5075 and *S. aureus* UAMS-1 strains to represent a Gram negative and Gram positive pathogen for analysis. Untreated cells of the both *A. baumannii* and *S. aureus* showed a normal cell shape with an intact structure of the cell wall, inner membrane and outer membrane. The images of the *A. baumannii* samples treated with *L. rhamnosus* BS were markedly different to those of the untreated cells. Several BSLR treated *A. baumannii* cells had their membranes damaged at certain areas of the bacterial cell with accumulation of dense substance (Figure 5). Surprisingly we also found that *L. rhamnosus* BS seems to cause more damage specifically to the ends of the bacterial cell (Figure 5). *S. aureus* cells treated with *L. rhamnosus* BS exhibited profound structural differences when compared to untreated cells. Several cells were observed devoid of cell walls, a phenomenon called as “ghost” cells. The dark and light areas observed in the cells were indicative of high and low electron densities respectively. Several cells were found to contain septa when compared with the control samples (Figure 5). This may be due to BS interfering with the cell division process and this is probably indicative of one of the mechanisms of action of BSLR by inhibiting the cell division.

## Discussion

Here, we describe two cell-associated biosurfactants derived from probiotic lactobacilli bacteria that possess both antibacterial activities and inhibit biofilm formation by several important drug-resistant pathogens. Although some strains of lactobacilli are known to produce surfactants, to the best of our knowledge there are no reports on the abilities of *L. jensenii *and* L. rhamnosus* BS as anti-biofilm agents. The crude BS derived from aforementioned bacteria showed significant antimicrobial activities against *A. baumannii, E. coli,* MRSA and *S. aureus* at MIC concentrations ranging between 25 and 50 mg/ml. *L. jensenii* BS showed antimicrobial activity at 25 mg/ml against all three pathogens with killing that varied from 75-99.9%, and at 12.5 mg/ml, it showed 90-100% killing against MRSA. In addition, we observed similar antimicrobial activities from both BS with similar MIC concentrations against MDR *Klebsiella pneumoniae* (Table 2). In this study, we clearly showed the effect of BS on the bacterial cells by transmission electron microscopy (Figure 5). The bacterial cell membrane appears to be the target of BS activity and subsequent microbicidal activity of BS may be due to the leakage of cellular contents. BS that exhibit antimicrobial activity has been previously described [33-35], but to date there have been very few studies on the activity of BS isolated from lactobacilli [8,9,28,29,33]. The antimicrobial results generated from our studies were similar to the results from crude BS studies on *L. paracasei* ssp. *paracasei* A20 and *L. lactis,* which completely inhibited the growth of several micro-organisms at concentrations ranging between 25 and 100 mg/ml [8,9,33]. In addition, BS exhibited excellent anti-adhesive properties against *S. aureus, A. baumannii* and *E. coli,* as evidenced in our adhesion based assays.

Next, the biofilm inhibition and dispersal capability of BS against the above mentioned three pathogens was evaluated. The crude BS isolated in this study exhibited very good anti-biofilm activities against select microorganisms. In the past, several studies have documented the anti-biofilm activity of BS isolated from different bacteria [19-21]. Preformed biofilms of all the three bacteria tested in microtiter plate wells were effectively disrupted by the BS. The BS dispersed all three bacteria when exposed for longer durations. The inhibition of biofilms using surfactants has been reported previously [12]. In addition, BS have been shown to disperse biofilms of *Bordetella bronchiseptica* and *B. pumilus*[19,22]. The increased anti-biofilm and dispersal ability of the BS currently being investigated may be due to the two important properties that it displays, namely, antimicrobial and surfactant activity. Previous studies with a BS isolated from *Lactobacillus spp*. have only demonstrated its antimicrobial and anti-adhesive properties [33]. Here we report for the first time BS isolated from lactobacillus also displaying anti-biofilm properties. Both *L. jensenii* and *L. rhamnosus* BS were able to significantly reduce biofilm development by *A. baumannii*, *E. coli* and *S. aureus.*

The use and applications of BS in the medical field has increased considerably in the last years. The impact of BS in bacterial adhesion and desorption has been widely discussed, and subsequent adsorption of these BS to solid abiotic surfaces could prove an effective strategy to reduce bacterial adhesion, thereby combating microbial colonization in both medicine and industry [7,30,36,37]. The anti-biofilm and anti-adhesive activities of the BS observed against several pathogens in our studies opens up the possibility of using them to coat a variety of medical surfaces to drastically reduce microbial colonization.

In conclusion, in this work we have demonstrated the antimicrobial, anti-biofilm, and anti-adhesive properties of the crude BS isolated from *L. jensenii* against several pathogenic MDR bacteria. We also observed that addition of both BS at effective concentrations to eukaryotic cells resulted in low cytotoxicity (Figure 4), suggesting the safety of these compounds for topical delivery. Biofilm encroachment on biomaterial is an extremely important concern post-surgery. Hence, our results open the possibility of using BS-modified materials for the construction of biofilm-resistant medical implantable devices, given its broad-spectrum activity against both Gram-positive and Gram-negative bacterial strains. BS exhibits potential as a new therapeutic strategy to inhibit biofilm formation. Because the increased prevalence of antibiotic-resistant bacteria generates a need for alternate and novel strategies to combat biofilms, we believe that co-administering antibiotics with anti-biofilm agents that possess surface activities such as BS may form the basis of future clinical protocols against biofilm-based infections.

Currently, experiments are underway to characterize the crude fractions of the surfactants by high performance liquid chromatography. We have identified certain fractions that exhibit excellent antimicrobial activities against *A. baumanni*, *S. aureus*, *K. pneumoniae* and *E. coli*. These fractions are being further analyzed by mass spectrometry and Matrix assisted laser desorption/ionization- time of flight (MALDI-TOF) to identify the active component/s.

## Conclusion

The BS produced by *L. jensenii* and *L. rhamnosus* showed antiadhesive, antimicrobial and antibiofilm activities against several MDR bacteria, such as *E. coli*, *A. baumannii* and *S. aureus*, which are prominent biofilm formers on wounds, medical implants and industrial surfaces. Antiadhesive and antimicrobial activities were seen between 25-100 mg/ml. MBEC based biofilm assays confirmed the inhibitory action of BS on biofilm development at concentrations ranging 25-50 mg/ml. In addition, BS dispersed preformed biofilms of *A. baumannii* and *S. aureus* at concentrations ranging 50-100 mg/ml. Due to its surface tension reduction properties, BS can be used to coat medical surfaces to prevent microbial colonization by variety of bacteria causing indwelling device associated infections.

## Methods

### Strains and growth conditions

*L. jensenii* 25258 and *L. rhamnosus* 7469 were purchased from ATCC (ATCC, Manassas, VA, USA). The strains were stored at -80°C in MRS broth (Difco, Sparks, MD, USA) containing 20% (v/v) glycerol*.* Multi-drug-resistant test strains were stored at −80°C in the appropriate media with 15% (v/v) glycerol until use. *E. coli* strains EC433*,* EC438*, A. baumannii* strains AB5075, AB5711 were cultured in LB broth and *S. aureus* clinical isolate 243 (MRSA) and UAMS-1 in trypticase soy broth. All the strains were grown at 37°C for 4 h in the appropriate media, and were washed in PBS 2X and resuspended in PBS to the appropriate OD at 600 nm for testing.

### BS production and isolation

To isolate cell-associated BS, the following protocol was pursued according to previously published methods used by several other investigators for lactobacilli BS [8,10,33]. For crude BS production by *L. jensenii* 25258*,* 1200 ml of MRS culture broth was inoculated with 10 ml of an overnight culture of *L. jensenii* and incubated for 48 h at 37°C at 120 RPM on a shaker. For *L. rhamnosus,* 1200 ml culture of MRS broth was inoculated with 10 ml of an overnight culture and incubated for 48 h at 33°C without shaking. After 48 hours, cell pellets were collected by centrifugation (10000 × g, 10 min, 10°C), washed twice in demineralized water, and re-suspended in 100 ml of PBS. This solution was gently stirred at room temperature for 2 h to release the cell-bound BS. After 2 hours, bacteria were removed by centrifugation and the supernatant liquid was collected by filtering through a 0.22 um filter. The filtered sterile supernatant was lyophilized. The freeze-dried BS was stored at -20°C, and resuspended in deionized water at 100 mg/ml (w/vol).

### Antimicrobial assay with BS

The antimicrobial activities of *L. jensenii* and *L. rhamnosus* crude BS against several pathogens were determined in 96-well tissue culture plates by a modified microdilution method [33]. Briefly, 100 μl of sterile, double-strength MHB broth was placed into the first column of the 96-well microplate and 100 μl of sterile, single-strength MHB broth in the remaining wells. Subsequently, 100 μl of BS solution in PBS (100 mg/ml) was added to the first column of the microplate and mixed with the medium; this resulted in a BS concentration of 50 mg/ml, and 100 μl was transferred serially to the subsequent wells, resulting in two-fold dilutions. Columns without BS served as positive growth controls. All the wells were inoculated with 100 μl of 10^8^ CFU from each of the test strains from a log phase culture. Microtiter plates were covered and incubated for 24 h under the appropriate growth conditions for each microorganism. Three independent preparations of crude BS were tested in duplicate. The contents of each well were plated onto LB agar plates with appropriate dilutions, and CFU were enumerated the next day. Percent killing was calculated as 1- (treated/control × 100).

### Oil spreading assay to determine surfactant activity

For the oil spreading assay, 50 ml of demineralized water was added to a 150 mm diameter Petri dish and 20 μl of motor oil was added to the surface of the water. Ten microliters of crude BS from either *L. jensenii* or *L. rhamnosus,* dissolved in deionized water was then added to the surface of the oil at concentrations ranging from 6.25 to 50 mg/ml. Surfactin was used as a positive control at the same concentrations, and a negative control was included with PBS. The diameters of clear zones of triplicate assays from the same sample were determined.

### Cytotoxicity assay

The human lung epithelial cell line (A549) was used in this study. The cytotoxicity of the crude BS from both *Lactobacilli* was evaluated on eukaryotic cells by the release of lactate dehydrogenase (LDH) and total cell number assay. The LDH cytotoxicity assay was performed according to the manufacturer’s guidelines (CytoTox 96 Non-Radioactive Cytotoxicity Assay, Promega, Madison, WI, USA). After the addition of the crude BS at different concentrations for 24 h, the cell culture medium was collected for LDH measurement after lysis of cells. An aliquot of 50 μl cell medium was used for LDH activity analysis and the absorption was measured using a UV–visible spectrophotometer. Percentage cytotoxicity was calculated as the percentage of LDH released compared to untreated cells. All experiments were repeated three times, each in triplicate.

### Assessment of biofilm formation

Biofilm formation was measured under two static conditions using a quantitative crystal violet assay. BS was added to the wells that contained the media and bacterial cells. BS were used at 25-50 mg/ml concentrations in final total volume not exceeding 165 μl, the volume at which the biofilms develop well in the MBEC biofilm assay. Biofilms of *A. baumannii* were developed on polystyrene 96-well and MBEC (Biosurface Technologies, Bozeman, MT, USA) as described previously by Sambanthamoorthy, 2012 [38]. Briefly, cultures grown overnight were standardized to an OD_595_ of 0.05 and 165 μl was transferred to the wells of a 96-well polystyrene microtiter plate, and the MBEC lid was placed on top of the wells. BS were added concurrently to the wells and biofilms were grown on the pegs under shaking conditions for 24 h. The lid was removed and the pegs were gently washed twice with 200 μl of PBS to remove non-adherent cells. Adherent biofilms on the pegs were fixed with 200 μl of 100% ethanol prior to staining for 2 min with 200 μl of 0.41% (wt/vol) crystal violet in 12% ethanol (Biochemical Sciences, Swedesboro, NJ, USA). The pegs were washed several times with PBS to remove excess stain. Quantitative assessment of biofilm formation was obtained by the immersion of pegs in a sterile polystyrene microtiter plate which contained 200 μl of 100% ethanol and incubation at room temperature for 10 min before the absorbance at 595 nm was determined. Three independent experiments were performed for each of these assays. Biofilms of *S. aureus* were developed on polystyrene 96-well plates and evaluated against the BS as previously described [39].

### Biofilm dispersal

To determine if the BS could disperse preformed biofilms, bacterial biofilms were established as previously described [38,40]. Briefly, established biofilms were exposed to varying concentrations of the BS in fresh media for short time intervals. Adherent biofilms on the pegs were fixed with 200 μl of 100% ethanol prior to staining for 2 min with 200 μl of 0.41% (wt/vol) crystal violet in 12% ethanol (Biochemical Sciences, Swedesboro, NJ, USA). Quantitative assessment of biofilm formation was obtained by the immersion of pegs in a sterile polystyrene microtiter plate which contained 200 μl of 100% ethanol; the absorbance at 595 nm was determined using a SpectraMax M5 microplate spectrophotometer system. Results were interpreted by the comparison of BS on treated biofilms to untreated biofilms. Experiments were performed in triplicate and three independent experiments were performed for each of these assays.

### Adherence of *A. baumannii, E. coli* and *S. aureus* to abiotic surfaces

An initial adherence assay was used to measure the impact of BS on the surface binding capacity of *A. baumannii, E. coli* and *S. aureus*. The assay was performed by modifying a microtiter biofilm assay as described previously [39]. Briefly, overnight cultures of *A. baumannii* and *S. aureus* test strains were diluted to an absorbance value of 0.05 at 595 nm in fresh medium, and 200 μl was added to each well (polystyrene pre-coated with human plasma) in triplicate. This was followed by adding BS at relevant concentration to be tested into the wells. Following 1 h incubation at 37°C, the microtiter wells were washed three times with PBS. Adherent cells were then fixed with 200 μl of 100% ethanol for 10 min. The ethanol was removed and the wells were air dried for 2 min. Adherent cells were stained for 2 min with 200 μl of 0.41% crystal violet (w/v in 12% ethanol), then washed three times with PBS. The wells were allowed to dry and then eluted with ethanol. Absorbance readings were made at 595 nm using a SpectraMax M5 microplate spectrophotometer system (Molecular Devices, Sunnyvale, CA, USA). Experiments were performed in triplicate and three independent experiments were performed for each of these assays.

### Transmission electron microscopy

A log phase culture of *A. baumannii* and *S. aureus* in separate tubes containing LB broth was split into 1.5 ml aliquots. The cells were collected by centrifugation (10000 × g, 5 min) and resuspended in PBS (Sigma-Aldrich, St. Louis, MO, USA). Four samples were prepared; two untreated, two treated with BSLR (4X MIC). The samples were incubated at 37°C for 3 h. The cells were collected by centrifugation (10000 × g, 5 min) to aspirate the supernates. To fix the cells, 4% glutaraldehyde was added to the pellet and the samples were incubated at 4°C for 1 h. The cells were collected by centrifugation (10000 rpm, 5 min) and washed twice with 0.1 M phosphate buffer. To postfix the cells, 1% osmium tetraoxide was added and the samples were left at room temperature for 1 h. The samples were dehydrated with graded ethanol solutions (50% ethanol for 15 min, 70% ethanol for 15 min, 95% ethanol for 15 min, and 100% ethanol for 30 min), embedded in epon and left to polymerize for 24 hrs. From each sample 10 thin slices (approximately 100 nm) were cut with Leica ultra cut UCT (Leica, Buffalo grove, IL, USA). Each of these sections was examined with a JEOL 1400 transmission electron microscope (JEOL, Peabody, MA, USA).

## Abbreviations

BS: Biosurfactants; MDR: Multidrug-resistant; MRSA: Methicillin resistant *Staphylococcus aureus*; EPS: Extracellular polysaccharide; MBEC: Minimum biofilm eradication concentration.

## Competing interests

The authors declare that they have no competing interests.

## Authors’ contributions

KS designed and developed the assays for adhesion, biofilm inhibition and dispersion. KS also drafted the manuscript. XF carried out the antimicrobial and toxicity assays. SP and RP isolated the BS and performed the oil spreading assay. CP conceived of the study, participated in its design and coordination, and also drafted the manuscript. All authors read and approved the final manuscript.
